# 3.5Å cryoEM Structure of Hepatitis B Virus Core Assembled from Full-Length Core Protein

**DOI:** 10.1371/journal.pone.0069729

**Published:** 2013-09-06

**Authors:** Xuekui Yu, Lei Jin, Jonathan Jih, Chiaho Shih, Z. Hong Zhou

**Affiliations:** 1 Department of Microbiology, Immunology and Molecular Genetics, University of California Los Angeles, Los Angeles, California, United States of America; 2 California NanoSystems Institute, University of California Los Angeles, Los Angeles, California, United States of America; 3 Institute of Biomedical Sciences (IBMS), Academia Sinica, Taipei, Taiwan; University of Leeds, United Kingdom

## Abstract

The capsid shell of infectious hepatitis B virus (HBV) is composed of 240 copies of a single protein called HBV core antigen (HBc). An atomic model of a core assembled from truncated HBc was determined previously by X-ray crystallography. In an attempt to obtain atomic structural information of HBV core in a near native, non-crystalline environment, we reconstructed a 3.5Å-resolution structure of a recombinant core assembled from full-length HBc by cryo electron microscopy (cryoEM) and derived an atomic model. The structure shows that the 240 molecules of full-length HBc form a core with two layers. The outer layer, composed of the N-terminal assembly domain, is similar to the crystal structure of the truncated HBc, but has three differences. First, unlike the crystal structure, our cryoEM structure shows no disulfide bond between the Cys61 residues of the two subunits within the dimer building block, indicating such bond is not required for core formation. Second, our cryoEM structure reveals up to four more residues in the linker region (amino acids 140-149). Third, the loops in the cryoEM structures containing this linker region in subunits B and C are oriented differently (~30° and ~90°) from their counterparts in the crystal structure. The inner layer, composed of the C-terminal arginine-rich domain (ARD) and the ARD-bound RNAs, is partially-ordered and connected with the outer layer through linkers positioned around the two-fold axes. Weak densities emanate from the rims of positively charged channels through the icosahedral three-fold and local three-fold axes. We attribute these densities to the exposed portions of some ARDs, thus explaining ARD’s accessibility by proteases and antibodies. Our data supports a role of ARD in mediating communication between inside and outside of the core during HBV maturation and envelopment.

## Introduction

Hepatitis B virus (HBV) infection is the leading cause of liver cirrhosis and hepatocellular carcinoma in humans, resulting in over a million deaths worldwide each year [1–3]. HBV is a member of orthohepadnaviruses within the family *Hepadnaviridae* [4]. The virus consists of a host-derived lipid envelope containing surface proteins known as HBV surface antigen (HBs), a nucleocapsid containing a core protein known as HBV core antigen (HBc) and a partially double-stranded DNA genome [4,5].

In addition to HBs, HBc, a third antigen, HBV e antigen (HBe), also exists. While both HBs and HBc are structural components of infectious HBV, HBe is a non-structural protein. Though the exact function of this non-structural protein is unknown, it is thought that HBe may play a role in suppressing immune response to HBV infection [6,7]. HBe shares an identical 1-149 amino acid sequence with HBc, and while lacking the basic arginine-rich domain (ARD) at the C-terminus of HBc, mature HBe contains an additional 10-amino acid prepeptide at its N-terminus [8,9]. Although HBc and HBe share most of their amino acid sequences, they exhibit distinct antigenic properties. HBc can assemble into two different morphologies (T=3 or T=4 icosahedral symmetry containing 180 or 240 HBc subunits, respectively) both in infected cells and in cell-free, in vitro assembly systems [10–12]. While HBc protein is able to self-assemble into such cores, HBe protein lacks such ability to form stable particles.

HBc consists of two distinct domains: an N-terminal assembly domain (residues 1-149) and a basic, C-terminal ARD (residues 150-183 or 150-185, depending on the strain). While the assembly domain forms the icosahedral shell, the ARD is involved in packaging viral pregenomic RNA (pgRNA) [13,14].

HBV core has been subjected to extensive structural studies by cryo electron microscopy (cryoEM) [11,12,15–17] and by X-ray crystallography [18]. Notably, the structures of recombinant particles assembled from truncated HBc containing only the assembly domain (i.e., truncated core) have been determined first to sub-nanometer resolutions by cryoEM [15,16] and subsequently to 3.3Å resolution by X-ray crystallography [18]. These structures have resolved the fold of the assembly domain with four long helices (>3 helical turns each). A dimer of HBc molecules is formed through a four-helix bundle which contains two long helices from each monomer. This HBc dimer acts as the building block for core assembly. In particular, the crystal structure revealed a disulfide bond between the two Cys61 residues within the helix bundle of the dimer building block. Other studies with biochemical and biophysical observations have also shown the existence of Cys61–Cys61 inter-molecular disulfide bond and suggested a role of it in core stabilization [14,19–24]. In contrary, one biochemical study showed that intracellular HBV core particles did not contain any disulfide bond [25], raising doubts about the physiological relevance of the disulfide bonds observed in the other studies.

Here, by using single particle cryoEM, we determined the three-dimensional (3D) structure of HBV cores assembled from full-length HBc (i.e., full-length core) to 3.5Å resolution and built an *ab initio* atomic model, revealing differences from the crystal structure of the truncated core. Remarkably, our cryoEM structure and biochemical data both show no disulfide bond formation, thereby refuting a role of Cys61–Cys61 inter-molecular disulfide bond in HBV core formation. Our results point to a role of the conserved Cys61 in HBe folding and support an intra-molecular disulfide bond-based mechanism for this folding. Additionally, weak densities attributed to the externally exposed portions of some ARDs provide an explanation for how the HBc ARD tails in the core interior can be accessible to proteases and antibodies [26,27]. We suggest a mechanism through which ARD functions in the processes of HBV core intracellular cytoplasmic trafficking, maturation and envelopment through shuttling between interior and exterior of the core [28].

## Results

### CryoEM structure determination of the HBV full-length core

**Figure 1:**
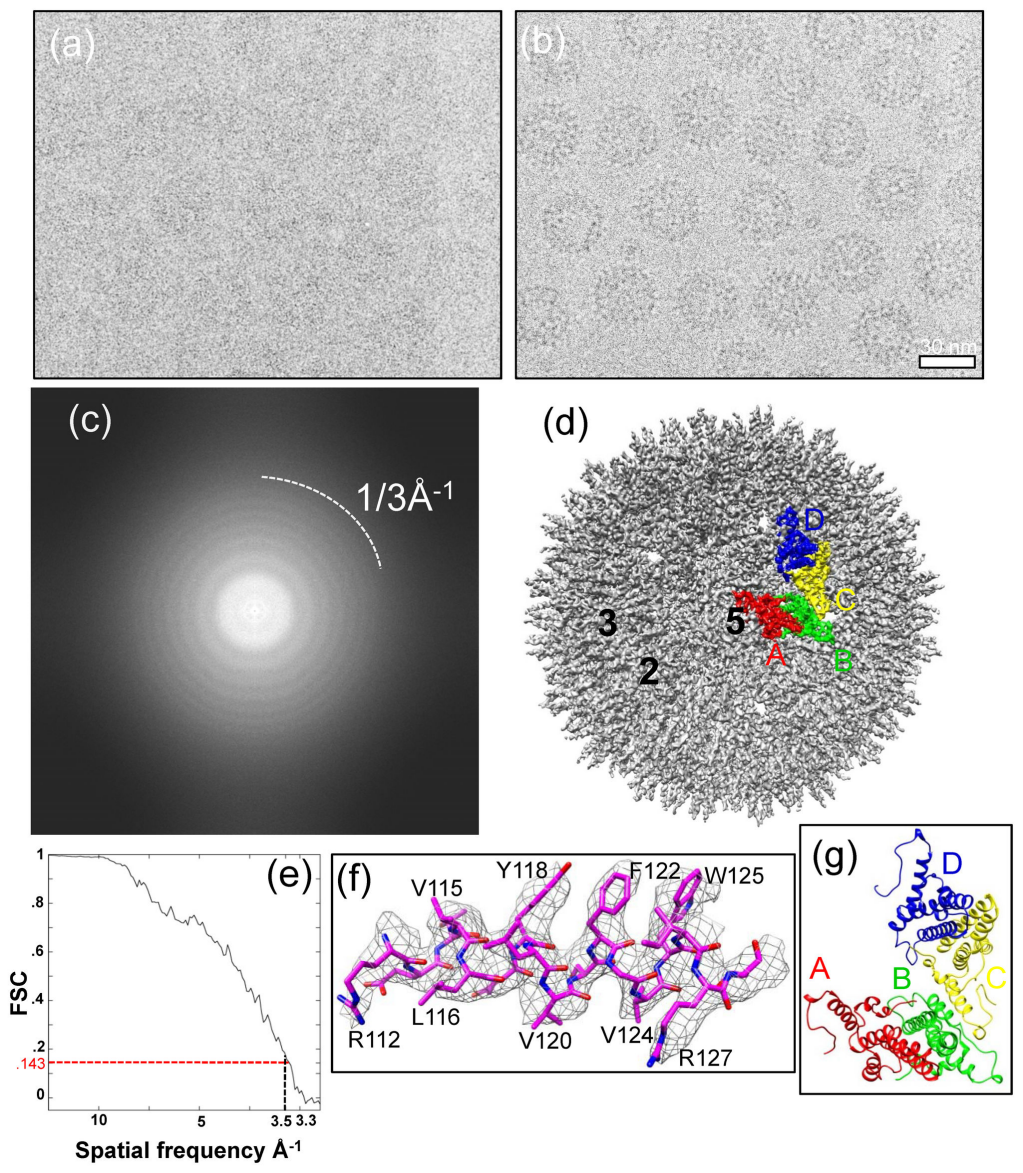
CryoEM and 3D reconstruction of hepatitis B virus (HBV) core assembled from full-length HBV core proteins at 3.5Å resolution. (a, b) Representative focal-pair cryoEM images of core particles embedded in a thin layer of vitreous ice, recorded on Kodak So 163 film in an FEI Titan Krios cryo electron microscope operated at 300kV at liquid-nitrogen temperature. (c) Fourier transform of the cryoEM image in (a), showing contrast transfer function rings visible up to 1/3Å^-1^. (d) Shaded surface representation of the core reconstruction at 3.5Å resolution as viewed along a five-fold axis. One asymmetric unit is segmented and color-coded. The A, B, C, and D subunits that form the asymmetric unit are colored in red, green, yellow, and blue, respectively. The two-fold, three-fold, and five-fold axes are indicated by the numbers 2, 3, and 5, respectively. (e) Resolution assessment of HBV 3D reconstruction based on the 0.143 criterion of reference-based Fourier shell correlation coefficient, showing that the effective resolution is ~3.5Å. (f) Atomic model (stick) of an α-helix is superimposed in its density map (mesh). (g) An atomic model of an asymmetric unit of the T=4 HBV core derived from the cryoEM structure viewed along a five-fold axis. The A, B, C, and D subunits that form the asymmetric unit are color-coded in red, green, yellow, and blue, respectively.

Previous high resolution X-ray crystallography study of HBV core has relied on capsid-like particles assembled from recombinant core protein truncated at amino acid 149, which yielded crystals that diffracted to a resolution of 3.3Å [18]. In this study, we aimed to determine the structure of HBV cores assembled from full-length HBc. We obtained highly purified full cores for cryoEM imaging (See Methods). Our cryoEM images (Figure 1a-b), captured in a FEI Titan Krios cryo electron microscope operated at 300 kV, contain data up to 3Å resolution (Figure 1c). We selected 191 “good” cryoEM micrographs (those without noticeable specimen drift and charging) from a total of 397 micrographs for in-depth data processing. The power spectra of these micrographs all show visible CTF rings beyond 1/4.5 Å^-1^ (e.g., Figure 1c).

The final density map (Figure 1d) was reconstructed by combining 8,093 close-to-focus particle images selected from 84,458 particle images. We estimate the effective resolution of the final reconstruction to be 3.5Å, based on the Fourier shell correlation coefficient (FSC) criterion (Figure 1e) defined by Rosenthal and Henderson [29].

**Figure 2:**
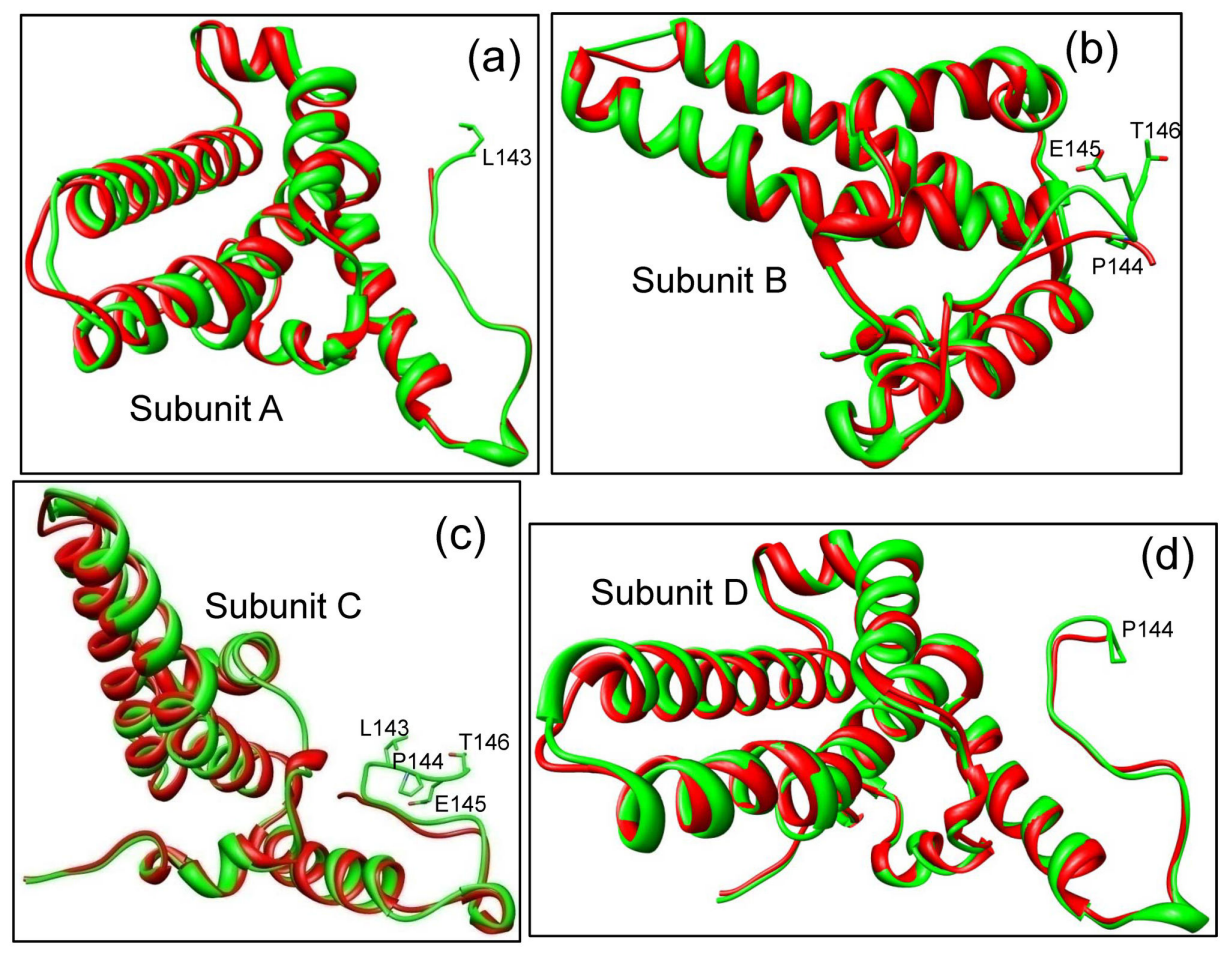
Comparisons between corresponding cryoEM structures (green) and crystal structures (red) by superimposition. (a–d) The cryoEM-derived structures of the four subunits, namely subunits A (a), B (b), C (c), and D (d), from an asymmetric unit are similar to their crystal structure counterparts. Side chains are only shown for those residues that were resolved in the cryoEM structure of full-length core, but not in the crystal structure.

Side chain densities of the HBc amino acid residues are readily recognizable in our 3.5Å cryoEM density map (Figure 1f). Using atomic modeling tool *COOT* [30], we built a full atomic model for the HBV core by tracing the backbone and side chain densities revealed in the cryoEM reconstruction (Figure 1f). Due to the T=4 icosahedral symmetry of HBV core, each asymmetric unit of the full-length core contains four structurally unique, non-symmetrically related HBc molecules, denoted as A, B, C, and D (Figure 1g). The atomic model of the asymmetric unit contains residues 1-143 of molecule A, residues 1-146 of molecules B and C, and residues 1-144 of molecule D (Figure 1g and Figure 2). Densities corresponding to the C-termini of these molecules are only visible at lower resolution and were not modeled.

### The outer layer of the full-length core: an atomic model of the N-terminal assembly domain

Unlike the single-shelled structural organization of truncated HBV core, which were determined previously by cryoEM [15,16] and X-ray crystallography [18], our cryoEM reconstruction of full-length HBV core revealed a ~360Å icosahedral capsid with a double-layer structure. The outer layer is icosahedrally ordered and has an overall appearance indistinguishable from that of the truncated core. The structures of the four HBc molecules in each asymmetric unit closely resemble each other, with root mean square deviations (rmsd) of 0.868Å (A vs. B), 0.7Å (A vs. C), and 0.632Å (A vs. D) (Figure 1g). Each monomer is composed of primarily helices and has a characteristic hairpin of long α helices, as previously revealed by X-ray crystallography [18].

Overall, the atomic models of the four HBc molecules derived from our cryoEM reconstruction are similar to their corresponding models determined by X-ray crystallography, with rmsd values of 0.737Å, 0.726Å, 0.667Å, and 0.677Å for the Cα atoms of molecules A, B, C, and D, respectively. However, at the local level, the cryoEM models differ from the X-ray models in several significant ways. First, the cryoEM structures of molecules A, B, C, and D have 1-4 more residues resolved (the X-ray structure of truncated HBV core contains 1-142 residues of molecules A and C, and 1-143 residues of molecules B and D). These newly resolved residues in the cryoEM model are located at the C-terminal linker region (Figure 2), where the N-terminal assembly domain connects to the C-terminal ARD in the full-length protein. Second, among the four conformers of the asymmetric unit, molecules B and C both have their C-terminal segments oriented differently in the cryoEM structures of the full-length core as compared to those solved by X-ray crystallography of the truncated core (Figure 2b-c). These differences are most likely due to interactions between the ARD domain and the assembly domain in the full-length core, as described below.

**Figure 3:**
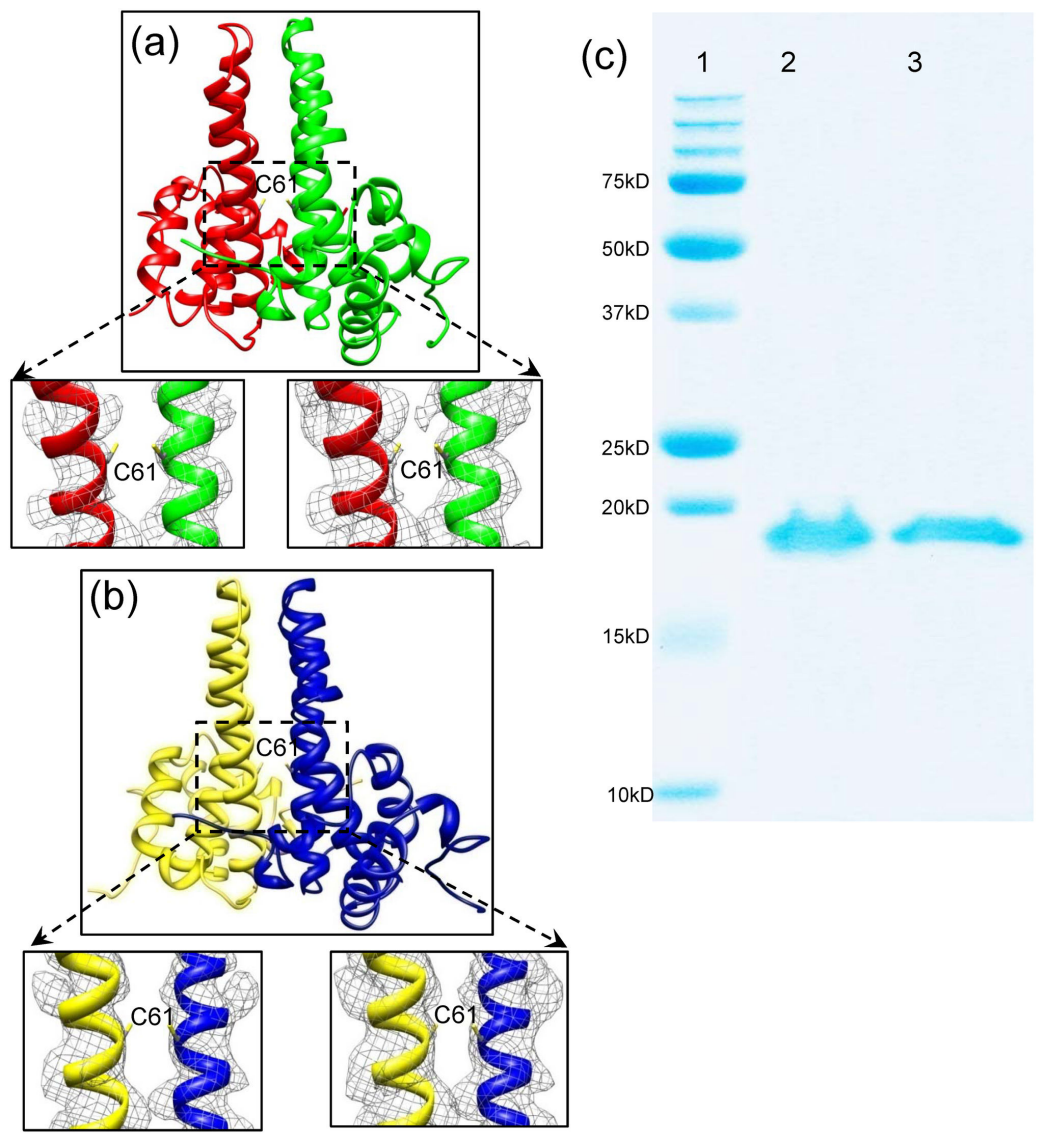
The HBV core dimer structures and SDS-PAGE analysis of the HBV core protein. (a) The dimer structure of subunits A (red) and B (green) shows no disulfide bond formation between the Cys61 residues of the two monomers (Insets). Density maps superimposed with atomic models of two helices from the boxed region, showing no density that can be attributed to disulfide bond formation between the two interacting monomers in this region. The density maps are contoured at 4.5σ (left inset) and 3σ (right inset) above the mean. (b) The dimer structure of subunits C (yellow) and B (blue) also show no disulfide bond formation between the Cys61 residues of the two monomers based on the cryoEM density map (Bottom insets). Density maps superimposed with atomic models of two helices from the boxed region, showing no density that can be attributed to disulfide bond formation between the two interacting monomers in this region. The density maps are contoured at 4.5σ (left inset) and 3σ (right inset) above the mean. (c) SDS-PAGE analysis of freshly-purified HBV core reveal no disulfide bond formation between core subunits. Lane 1: molecular-weight standards from Bio-Rad. Lane 2: HBV core boiled for 5 minutes under non-reducing condition; no dimer band was observed. Lane 3: HBV core boiled for 5 minutes under reducing condition with 5% (v/v) β-mercaptoethanol. A 10µg protein sample per lane was loaded and visualized by Coomassie Blue staining.

During the HBV core assembly process, two monomers interact with each other to form a dimer. 120 dimers then assemble into a T=4 core. Each asymmetric unit of the core contains two dimers: an A-B dimer and a C–D dimer, In the X-ray structure, a disulfide bond was observed between the two Cys61 residues of the interacting monomers within the A-B dimer or the C–D dimer [18]. However, our cryoEM structure shows no density that can be attributed to disulfide bond formation between the two interacting monomers in this region of these dimers (Figure 3a-b), even if the cryoEM density map was displayed in a relatively low threshold (insets in Figure 3a-b), suggesting that Cys61 does not form intermolecular disulfide bonds in our freshly prepared sample used for cryoEM. Further SDS-PAGE analysis demonstrated the absence of any disulfide bond formation among the core proteins in our sample (Figure 3c).

### The inner layer of the full-length core and direct observation of the C-terminal ARD protrusion

**Figure 4:**
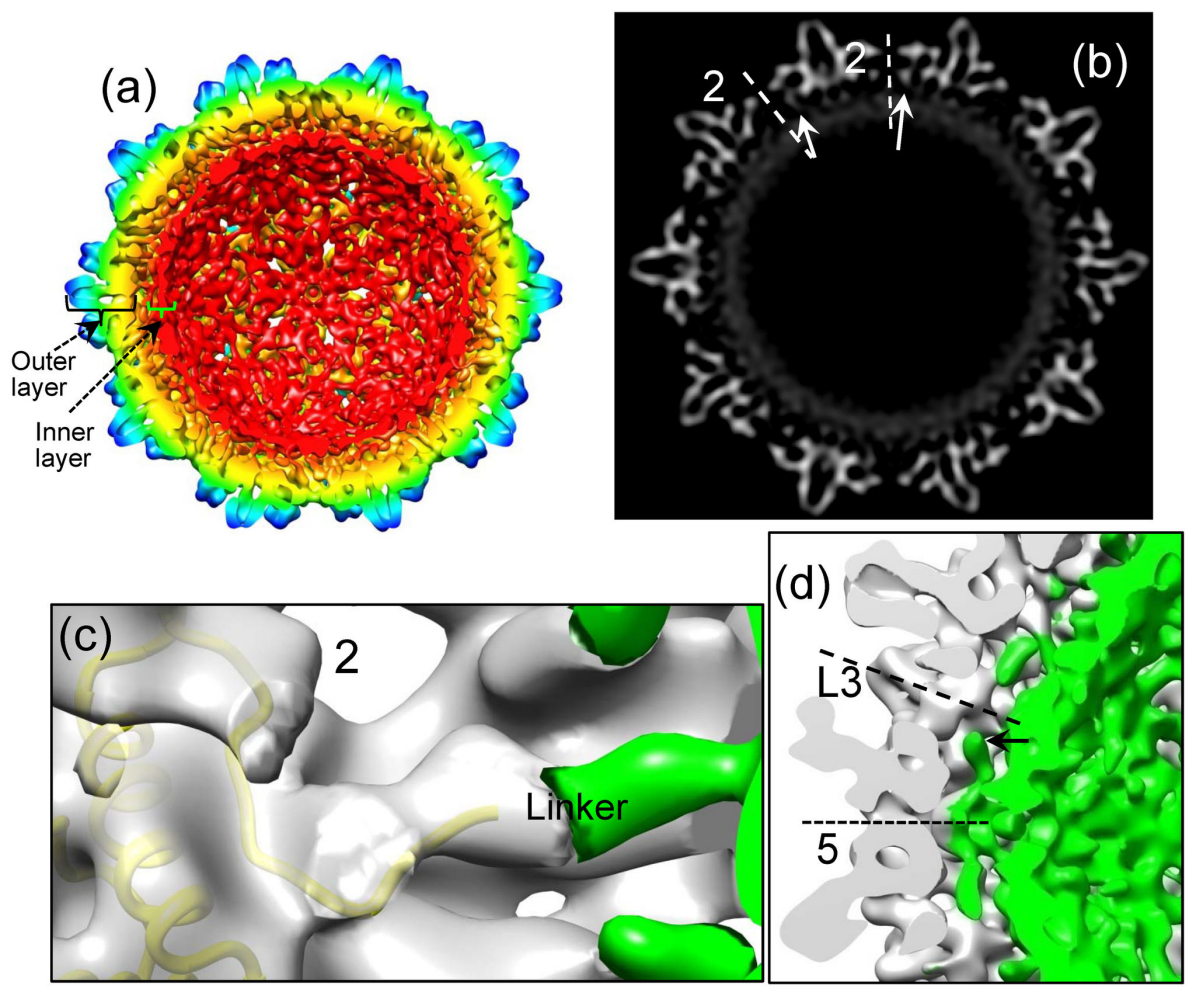
Maps of HBV core reconstruction filtered to 10Å resolution. All density maps are contoured at 2σ above the mean. (a) Inside view of the radially colored 3D reconstruction map, showing the full-length HBV core has a double-layer structure: an outer layer composed of the N-terminal core assembly domain (residues 1-149) and an inner layer composed of the basic C-terminal ARD (residues 150-185) and its bound RNAs. (b) A gray scale view of a central map slice from (a), showing links (arrows) connecting the outer layer and the inner layer. The links are located around 2-fold axes. (c) Density map superimposed with ribbon model of subunit C (yellow), showing the ordered C-terminal residues (residues 143-146) resolved in our cryoEM structure are superimposable with or close to the linker density around the 2-fold axis. The two-fold axis is indicated by the number 2. The ordered outer layer (transparent gray) is separated from the rest (green) of the structure by using atomic model. (d) A close-up view of density map, showing densities underneath one of the 12 vertices do not connect with either of the outer layer (gray) and the inner layer (green). Instead, these densities (arrow) extend toward the adjacent local 3-fold axes along the space between the outer and inner layers. The five-fold and local three-fold axes are indicated by the labels 5 and L3, respectively.

In addition to the outer layer composed of HBc’s N-terminal assembly domains as described above, our full-length core cryoEM structure also shows densities immediately inside the outer layer when the 3.5Å reconstruction was low-pass filtered to 10Å resolution. These densities forms a continuous inner layer located between radii 100–115 Å (Figure 4a-b). This inner layer can be interpreted as both packaged RNA molecules and the HBc’s ARD tails (residues 150-185) that are responsible for RNA binding. Numerous biochemical studies have shown that full-length cores assembled in *E. coli* can package RNA molecules non-specifically [31–35]. Though the inner layer of the core is clearly visible at a resolution of 10Å, it becomes less ordered at 3.5Å resolution (not shown), which suggests that the inner layer possesses a higher level of flexibility than the outer layer and/or is less ordered icosahedrally. The thickness of the inner layer is about 10-15Å, roughly corresponding to the diameter of a single-stranded RNA. Therefore, the packaged RNAs in this region are likely organized in a single molecular layer. Such single-layered organization of RNA may facilitate viral pgRNA sliding when it is reverse transcribed into DNA if the pgRNA molecule in the native core is organized in a similar way.

The outer and inner layers are connected by densities around the 2-fold axes (Figure 4a-b). These connecting densities are superimposable with, or in close proximity to, the newly resolved C-terminal residues of the assembly domain of molecules B, C and D in the cryoEM structure (Figure 4c), indicating that the connecting densities are the linker region (residues 140-149) of the core assembly domain.

**Figure 5:**
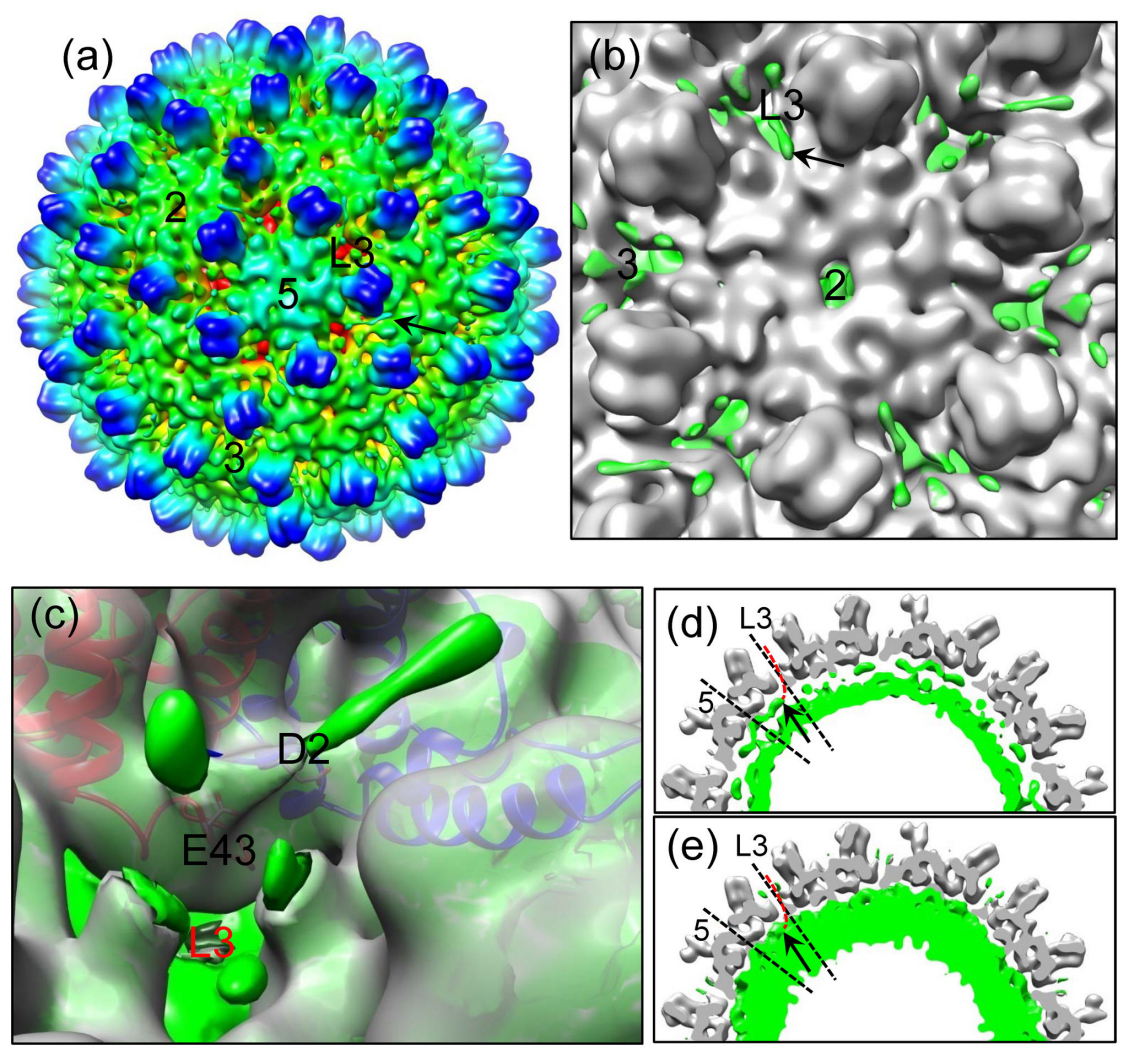
Direct observation of the HBc C-terminal ARD segments protruding outside the core. (a) Radially-colored, shaded surface representation of the HBV reconstruction filtered to 10Å resolution as viewed along a five-fold axis, showing some densities of the C-terminal ARD tails on the exterior of core (arrow). The two-fold, three-fold, local three-fold and five-fold axes are indicated by the labels 2, 3, L3, and 5, respectively. The density map is contoured at 1σ above the mean. (b) An enlarged view around a two-fold axis, showing a density ~20Å in length protruding outside the core through the local three-fold axis (arrow). The two-fold, three-fold, and local three-fold axes are indicated by the labels 2, 3, and L3, respectively. The ordered outer layer is colored in gray using atomic model, while the rest of density map is colored in green. The map is contoured at 1σ above the mean. (c) Density map (grey) superimposed with ribbon models of A (red) and B (blue) molecules, showing the positive-charged ARD density (green) interaction with the negative-charged D2 and E43 amino acids that line the local three-fold channel (L3). The ordered outer layer (transparent gray) is separated from the rest (green) of the structure by using atomic model. The density map is contoured at 1σ above the mean. (d, e) A slab (9.3 Å in thickness) across the local three-fold axis as viewed along a five-fold axis, showing densities (arrow) underneath one of the 12 vertices extending toward the adjacent local 3-fold axes and a possible pathway (red dotted line) for the ARD tail of molecule A protruding outside the core. While the ordered outer layer (gray) is contoured at 2σ above the mean in both maps, the rest (green) of the cryoEM map is contoured at 2σ in (d) and 1σ in (e), respectively. The five-fold and local three-fold axes are indicated by the labels 5 and L3, respectively.

Unexpectedly, the linker region residues in molecule A (i.e., the monomer around the 5-fold axes) do not form densities connecting the outer and inner layers. However, strong densities emanate from the 5-fold position to form five filamentous density protrusions in the space between the outer and inner layers (Figure 4d). Instead of being connected to the inner layer, the protruding end of this density extends towards the adjacent local 3-fold axis (arrow in Figure 4d). When the displaying threshold is decreased from 2σ to 1σ (standard deviation above the mean), densities protruding along holes at both the icosahedral three-fold and local three-fold axes can also be seen (Figure 5a,b). In particular, a filamentous density of ~20Å in length protrudes from the hole at each local three-fold position (Figure 5c). We interpret this filamentous density as part of the ARD tail, most likely of molecule A, the monomer near the 5-fold axis (see red dotted line in Figure 5d-e). Due to the strong positive charge of the ARD tail, it would be stabilized by the negative-charged amino acids, for example D2 and E43, which line the hole at the 3-fold positions (Figure 5c). This interpretation is consistent with previous biochemical results showing that the ARD tails of full-length cores were accessible to proteases and antibodies [26,27]. Holes at the two-fold axes were speculated as conduits for the ARD tails to reach outside from inside [18]. However, the full-length core structure reported here provides the first direct evidence supporting the use of three-fold related holes for the external exposure of the ARD (Figure 5).

## Discussion

Recently, by a heterokaryon and homokaryon analysis, Li et al. demonstrated that HBc protein could shuttle between nucleus and cytoplasm [28]. HBc ARD was shown to contain both nuclear export signals (NES) and a bipartite nuclear localization signal (NLS). Since the C-terminal tail of HBc has been mapped to the core interior by cryoEM [36], it is puzzling that the cellular importin machinery in the cytoplasm can see the NLS of HBc ARD buried inside the cores. As shown in Figure 5, we observed that the C-terminal ARDs can protrude outside the *E. coli*-expressed core through the global and local 3-fold axes. Therefore, C-terminal ARD could play an important role in mediating the communication between the core interior and exterior. In other words, the importin machinery can facilitate intracellular trafficking of HBc protein and particles via the recognition of the exposed NLS of HBc ARD. In HBV core maturation, particularly at the stage of plus-strand DNA synthesis, charge imbalance in the core interior could be gradually built up due to the increasing negative charge from the phosphodiester bond of the nascent plus-strand DNA elongation. According to the charge balance hypothesis [34,37,38], charge balance, core stability, and plus-strand DNA synthesis, can be maintained, if there is still a sufficient amount of serine dephosphorylation occurring at the HBc ARD. Perhaps, some of the exterior ARDs could re-enter the core interior to neutralize the increasing negative charges when the above-mentioned interactions between exterior ARDs and the negatively charged amino acids (Figure 5c) are disrupted by the sliding of minus-strand DNA template during the process of plus-strand DNA synthesis. As a consequence, the core would undergo conformational changes related to core maturation and envelopment [17].

The HBV genome has a very compact coding organization with four partially overlapping open reading frames (ORFs) that allow for the translation of seven proteins. The pre-C or C ORFs encode both the nucleocapsid structural protein HBc as well as the non-structural protein HBe. ORF C contains two in-frame start codons. HBc is the product of initiation from the internal start site, while the precore protein, an HBe precursor, results from initiation at the upstream AUG start codon. Although HBc and HBe share a large fragment of common sequence, and this common sequence includes the antigen epitopes of both HBc and HBe, HBc and HBe are distinctly recognized by antibodies [39,40].

HBc and HBe also share several cysteine residues, the only exceptions being that HBe contains an extra Cys(-7) residue relative to HBc and lacks the Cys185 residue found in HBc [41]. It has been proposed that Cys61 has a role in the stabilization of HBV core [14,19–24]. Indeed, both biochemical and structural data have shown that Cys61 residues from neighboring HBc monomers form an intermolecular disulfide bridge at the HBc dimer interface. However, our cryoEM structure of freshly prepared (<24 hours) HBV core sample clearly shows that Cys61 does not participate in disulfide bond formation—an observation further supported by SDS-PAGE analysis—thereby indicating that Cys61 plays no functional role in HBV core formation. We speculate that the previously identified Cys61 disulfide bridge in HBc dimers could be due to prolonged exposure to an oxidizing environment, e.g., oxygen dissolved in solution after the HBV core undergoes cytoplasmic release. Both the reducing environment and enzymatic activity of cellular cytoplasmic compartment prevent disulfide bond formation [42–44]. Indeed, proteins containing stable disulfide bonds are rarely found in the cytoplasmic compartments of most organisms. Because HBV core assembly and maturation take place exclusively in the cytoplasmic compartment, native HBV core should not contain any stable disulfide bonds. Our cryoEM and biochemical data of recombinant HBV cores are consistent with this notion and does not exclude the possibility that disulfide bond could form spontaneously during core envelopment or in the secreted virions [25].

It has been reported that conserved cysteines of HBc, including Cys61, are not required for assembly of replication competent core particles or for their envelopment [45]. This report was mainly based on functional study of HBc mutant containing Cys-to-Ser substitutions, and did not directly address the issue whether Cys61 can or cannot form disulfide bond. Our study here provides structural evidence for the lack of disulfide bond formation at Cys61 of freshly prepared HBc particles. Based on our observations (Figure 3), we propose that the conserved Cys61 residue functions in the formation of HBe. Indeed, the crystal structure of HBe has shown there are intramolecular disulfide bridges between Cys61 and Cys(-7) [46]. Interestingly, HBe is folded and processed within the lumen of the endoplasmic reticulum and secreted into the medium or blood circulation, which favors the formation of disulfide bonds.

## Methods

### Protein expression and HBV core purification

The gene for the full-length HBV core protein (subtype adw, GenBank accession # CAL29866), optimized for bacterial expression, was synthesized by GeneArt (Life Technologies Corporation, California). The amplified gene was double-digested with *Nde*I and *Bam*HI, and subsequently cloned into the expression vector pET-11a from Novagen. Transformed BL21 (DE3) cells were grown in LB medium with 100µg/ml of ampicillin at 37°C overnight. One liter of LB medium was inoculated with 20mL overnight culture. After IPTG was added into the culture medium with a final concentration of 0.2mM when A_600_ of medium reached 0.8, the bacterial cells were grown for another 4 hours to induce HBV core protein production.

Bacterial cells were pelleted by 4000*g* centrifugation and then re-suspended in 75mL of PBS buffer. 60mg of chicken lysozyme, 15mg of DNAse1, and two tablets of Roche protease inhibitor cocktail were added into the cell suspension. We disrupted the cells by a combination of lysozyme and 3 freeze/thaw cycles. The cell lysate was cleared by centrifugation at 10,000*g*. Solid ammonium sulfate was then added to the supernatant to 45% saturation. The mixture was left on ice for 2 hours to salt out the HBV core and then centrifuged at 10,000*g* for 15 minutes to pellet the HBV core. 6mL of PBS buffer was added to the pellet, and after incubation on ice for another 2 hours, nearly the entire pellet was dissolved. Residual un-dissolved aggregates were pelleted by centrifugation at 10,000*g* for 45 minutes. HBV core particles were further purified by 15-45% (buffered with PBS) sucrose gradient ultracentrifugation at 30,000 rpm for 2.5 hours using an SW41 rotor on a Beckman L8-80M ultracentrifuge. The band corresponding to HBV core was visualized by illuminating the gradient tube, after which the sample was carefully collected by side puncture. The purified HBV core sample was buffer-exchanged and concentrated to ~20mg/mL in PBS by ultra-filtration with a MW cutoff of 100kD (Millipore, USA).

### CryoEM imaging and 3D reconstruction

Purified HBV core particles were embedded in a thin layer of vitreous ice suspended across the holes of holey carbon films by plunge-freezing into liquid ethane. Before data collection, beam tilt was carefully minimized by coma-free alignment. Viral particle samples were kept at liquid-nitrogen temperature. CryoEM images were recorded on Kodak SO163 films at a dosage of ~25 electrons/Å^2^ on an FEI Titan Krios cryo electron microscope operated at 300kV and 75,000× nominal magnification with parallel beam illumination. Focal-pair images were recorded, with the first, close-to-focus micrograph at ~1.0 µm underfocus and the second, far-from-focus micrograph at ~3 µm underfocus. The films were digitized with a Zeiss SCAI microdensitometer scanner at a step size of 7 µm/pixel, corresponding to 0.933Å/pixel at the sample level.

We took a total of 397 micrographs and selected 191 micrographs that clearly showed signals beyond 1/4.5Å^-1^ in their spectra. Individual particle images (512x512 pixels) were first boxed out automatically by the *autoBox* program in the IMIRS package [47] and then followed by manual screening using the EMAN *boxer* program [48] to keep only the well-separated, contamination-free, intact RNA-containing particles.

The program CTFFIND [49] was used to determine the defocus value and astigmatism parameters for each micrograph. We determined particle orientation, center parameters, and performed subsequent 3D reconstruction with the IMIRS package [47], enhanced by the GPU-based *eLite3D* program for 3D reconstruction [50]. We considered astigmatism during CTF correction in the orientation/center refinement and 3D reconstruction steps. The final reconstruction was done using close-to-focus images only.

We assessed the effective resolution with the reference-based Fourier shell correlation (FSC) coefficient as defined by Rosenthal and Henderson [29]. The map was deconvolved by a temperature factor of 220Å^2^ (conventional definition) to enhance higher resolution features. The final reconstruction was low-pass filtered to a resolution of 3.5Å.

### Atomic model building, model refinement, and 3D visualization

At 3.5Å resolution, *ab initio* atomic model building was very straightforward. We first traced the peptide chain and built Cα models based on the clearly visible bumps of Cα atoms using the “Baton_build” utility of the atomic modeling program COOT [30]. Next, amino acid registration was accomplished solely by the visual identification of the densities of bulky side chains, which served as “landmarks” for sequence registration. Third, we built full atomic models in *COOT* with the help of *REMO* [51]. Rebuilding the model to fit the EM map was done manually with *COOT*, and the “regularize zone” utility of *COOT* was used to improve model stereochemistry.

These coarse full-atom models were then refined in a pseudocrystallographic manner using Phenix [52]. This procedure only improves atomic models and does not modify the cryoEM density map. Densities for individual proteins were segmented, put in artificial crystal lattices, and then used to calculate their structural factors. The amplitudes and phases of these structural factors were used as pseudo-experimental diffraction data for model refinement in Phenix. To improve the areas of interaction between different protein subunits, we put the refined structures of all four subunits from an asymmetric unit into a single coordinate file and pseudo-crystallographically refined them simultaneously with their non-crystallographic symmetry (NCS). This refinement process uses pseudo-experimental diffraction data generated from the 3.5Å cryoEM map of an asymmetric unit. The final R-factor (R-work) for the entire virus up to 3.5Å resolution was 0.26.

CryoEM reconstruction was visualized and segmented using *Chimera* [53]. All molecular figures were also prepared with *Chimera*.

### Access numbers

The cryoEM density map and atomic coordinate reported here are deposited in the EM Data Bank and the Protein Data Bank with accession codes EMD-2278 and 3J2V, respectively.
